# External sensory cueing on gait in Parkinson’s disease: a systematic review and network meta-analysis

**DOI:** 10.1007/s00415-026-13857-3

**Published:** 2026-05-22

**Authors:** Chernkhuan Stonsaovapak, Nantawan Koonalinthip, Prut Koonalintip, Chatkaew Pongmala, Pim Terachinda

**Affiliations:** 1 Department of Rehabilitation Medicine, Faculty of Medicine, Chulalongkorn University, Bangkok, Thailand; 2 Division of Neurology, Department of Internal Medicine, Faculty of Medicine, Prince of Songkla University, Songkhla, Thailand; 3 Functional Neuroimaging, Cognitive and Mobility Laboratory, Department of Radiology, University of Michigan, Ann Arbor, USA

**Keywords:** Parkinson disease, Cues, Feedback, Sensory, Gait speed, Spatiotemporal analysis

## Abstract

**Objective:**

This systematic review and network meta-analysis compares the individual and synergistic efficacy of external sensory cueing modalities to establish a treatment hierarchy for optimizing spatiotemporal gait parameters in individuals with Parkinson’s disease (PD).

**Methods:**

A systematic literature search was conducted across Scopus, PubMed, Cochrane CENTRAL, and ClinicalTrials.gov. Randomized controlled trials (RCTs) evaluating visual, auditory, somatosensory, or combined cueing modalities in individuals with PD were eligible. Trials were required to compare at least two active cueing modalities or an active modality against a control condition and report gait velocity and/or stride length.

**Results:**

Thirty-three RCTs (*n* = 1118 participants) were included. For gait velocity, somatosensory (MD = 0.12 m/s; 95% CI 0.04–0.19) and visual cues (MD = 0.11 m/s; 95% CI 0.02–0.20) demonstrated significant improvements over control. For stride length, visual (MD = 11.46 cm; 95% CI 6.40–16.51), somatosensory (MD = 10.74 cm; 95% CI 7.04–14.44), and auditory cues (MD = 5.25 cm; 95% CI 0.43–10.06) all yielded statistically significant benefits. *P*-score rankings identified somatosensory cues as the top-ranked modality for gait velocity (*P*-score = 0.71) and visual cues for stride length (*P*-score = 0.83). Global heterogeneity was substantial (*I*^2^ = 64–90%), and no significant publication bias was detected.

**Conclusions:**

External sensory cueing improves spatiotemporal gait parameters in PD, with somatosensory and visual cues emerging as the most effective modalities for enhancing gait velocity and stride length. Both modalities outperformed standalone auditory stimulation, providing a data-driven hierarchy to guide evidence-based cueing selection in clinical practice.

**Supplementary Information:**

The online version contains supplementary material available at 10.1007/s00415-026-13857-3.

## Introduction

As the second most prevalent neurodegenerative disorder, Parkinson’s disease (PD) is projected to see a sharp rise in global prevalence, significantly intensifying the public health burden [1]. A hallmark of PD is abnormal gait, characterized by reduced velocity, shortened step length, and increased variability [2]. Furthermore, patients often experience episodic gait dysfunction—unpredictable, intermittent disturbances primarily manifested as festination and freezing of gait (FOG) [2]. These impairments markedly elevate fall risk and foster a persistent fear of falling, initiating a restrictive cycle of physical inactivity that ultimately compromises patient quality of life [3].

Because gait abnormalities in PD are only partially responsive to pharmacological treatment, the integration of nonpharmacological strategies is essential. External cueing across various modalities—including visual cueing (VC), auditory cueing (AC), and somatosensory cueing (SC)—has proven effective in managing gait in PD by improving spatiotemporal parameters. Specifically, VC and AC enhance stride length, gait initiation, and turning quality by providing rhythmic pacing improving anticipatory postural adjustment, and reducing step variability [4]. The recent meta-analytic data further highlight that AC significantly increases gait velocity and cadence [5]. Although research into SC is expanding, definitive systemic evidence to confirm its efficacy remains limited as compared to other modalities [4]. The underlying mechanisms of cueing strategies remain not fully established. The current hypotheses primarily suggest that cueing compensates for subcortical deficits by engaging attentional pathways and bypassing impaired basal ganglia circuitry through the provision of external rhythmic stimuli [6–9]. Beyond individual applications, combining cueing strategies with physiotherapy yields superior improvements in gait outcomes compared to single-intervention approaches [10].

Although a growing body of evidence supports the role of external sensory cueing in patients with PD, the comparative efficacy of various cueing modalities remains insufficiently addressed. This systematic review and network meta-analysis (NMA) of randomized controlled trials (RCTs) aims to compare the efficacy of different external sensory cueing modalities, assessing both individual and synergistic effects. Furthermore, this analysis seeks to establish a treatment hierarchy to optimize the management of spatiotemporal gait parameters in the PD population.

## Methods

This systematic review and NMA was performed in adherence to the Preferred Reporting Items for Systematic Reviews and Meta-Analyses extension for Network Meta-Analysis (PRISMA-NMA) guidelines [11], and the protocol was prospectively registered with the International Prospective Register of Systematic Reviews (PROSPERO; registration number CRD420251273651). As this study synthesizes data from previously published literature and involves no direct patient interaction or collection of individual patient data, ethical approval was granted as exempt by the Institutional Review Board, Faculty of Medicine, Chulalongkorn University. Accordingly, patient consent was not applicable.

### Eligibility criteria

This review included RCTs involving individuals diagnosed with idiopathic PD, irrespective of the presence of FOG. Studies involving mixed neurological cohorts were included only if data for PD participants were extractable.

Eligible interventions comprised external sensory cueing or stimulation, specifically visual, auditory, somatosensory, or combined modalities, administered during gait or walking tasks. Trials combining different cueing modalities were coded as distinct intervention nodes to evaluate potential additive or synergistic effects.

Acceptable comparators included sham interventions, no intervention, or usual care. In designs where blinding via sham was unfeasible (e.g., exercise-based or device-assisted cueing), usual care or noncueing conditions were accepted as controls.

The outcomes of interest were key spatiotemporal gait parameters, including gait velocity and stride length. Only studies reporting quantitative outcome data suitable for meta-analysis were included.

Studies utilizing identical interventions in both arms were excluded. Nonrandomized designs, reviews, protocols, conference abstracts, and studies without full-text availability or sufficient extractable data were also excluded.

### Information sources and search strategy

A comprehensive search was executed across Scopus, PubMed, the Cochrane Central Register of Controlled Trials (CENTRAL), and ClinicalTrials.gov to locate relevant RCTs. The search strategy focused on three primary concepts: PD, external sensory cueing/stimulation, and gait. Databases were searched from inception through August 19, 2025, without publication year or language restrictions. Detailed search strings are available in “Supplementary file 1”.

### Study selection and data extraction

Covidence software [12] was utilized for record management and duplicate removal. Two reviewers independently screened titles and abstracts, resolving discrepancies through discussion or consultation with a third reviewer. Potentially eligible records underwent independent full-text review. Studies were excluded if data remained insufficient following attempts to contact authors. For non-English articles meeting the initial screening criteria, full-text versions were translated via online machine translation tools to determine final eligibility for inclusion.

Two reviewers independently extracted data regarding study design, sample size, participant characteristics, intervention specifics, and assessment timing.

The primary outcomes were gait velocity (m/s) and stride length (cm). Nonstandard units were converted to these metrics. Mean values and standard deviations (SDs) were extracted to calculate mean differences (MDs) and corresponding SDs [13]. Where the data were reported as medians and interquartile ranges (IQR), values were converted to means and SDs [14]. For studies reporting multiple follow-up time points, the assessment closest to the end of the last intervention session was selected to evaluate treatment effectiveness. In cases, where studies reported both comfortable and fast walking speeds, or both single- and dual-task conditions, data from the comfortable walking and single-task conditions were prioritized for inclusion in the analysis. Owing to data limitations, FOG outcomes were extracted and synthesized narratively as a post hoc analysis and were not included in the primary meta-analysis.

### Risk of bias assessment

The Cochrane Risk of Bias tool version 2 (RoB 2) was employed to assess bias in both parallel [15] and crossover trials [16]. The tool evaluates five domains: randomization, deviations from intended interventions, missing outcome data, outcome measurement, and selection of reported results. Studies were categorized as having low risk, some concerns, or high risk of bias, with visualizations produced via the *robvis* tool [17].

### Network geometry and statistical analysis

Network graphs were generated to visualize the evidence structure for both gait velocity and stride length. In these plots, nodes represent specific interventions, with sizes proportional to the total number of participants, while edges represent direct head-to-head comparisons, with thickness indicating the number of included studies.

Analyses were performed using a frequentist framework with the *netmeta* package in RStudio [18]. A random-effects model was employed for all comparisons to account for anticipated clinical and methodological diversity, such as variations in cueing delivery and medication states. Treatment effects for continuous outcomes (gait velocity and stride length) were calculated as MD with corresponding 95% confidence intervals (CIs). Intervention efficacy was ranked using *P* scores, which estimate the probability of one treatment being superior to others, with scores ranging from 0 to 1 (higher scores indicating greater effectiveness).

Statistical heterogeneity was quantified using the *I*^2^ statistic, with substantial heterogeneity defined as *I*^2^ ≥ 50% [19]. Consistency between direct and indirect evidence was evaluated at the global level using the design-by-treatment interaction model and at the local level using node-splitting analysis, with statistical significance set at *P* < 0.05. To ensure the robustness of the primary findings, several pre-specified sensitivity analyses were planned, including assessments based on risk of bias, small-study effects, and the exclusion of specific intervention modalities. Potential small-study effects and publication bias were further examined using comparison-adjusted funnel plots and formal statistical tests, including Egger’s and Thompson–Sharp tests.

## Results

### Study selection

**Fig. 1:**
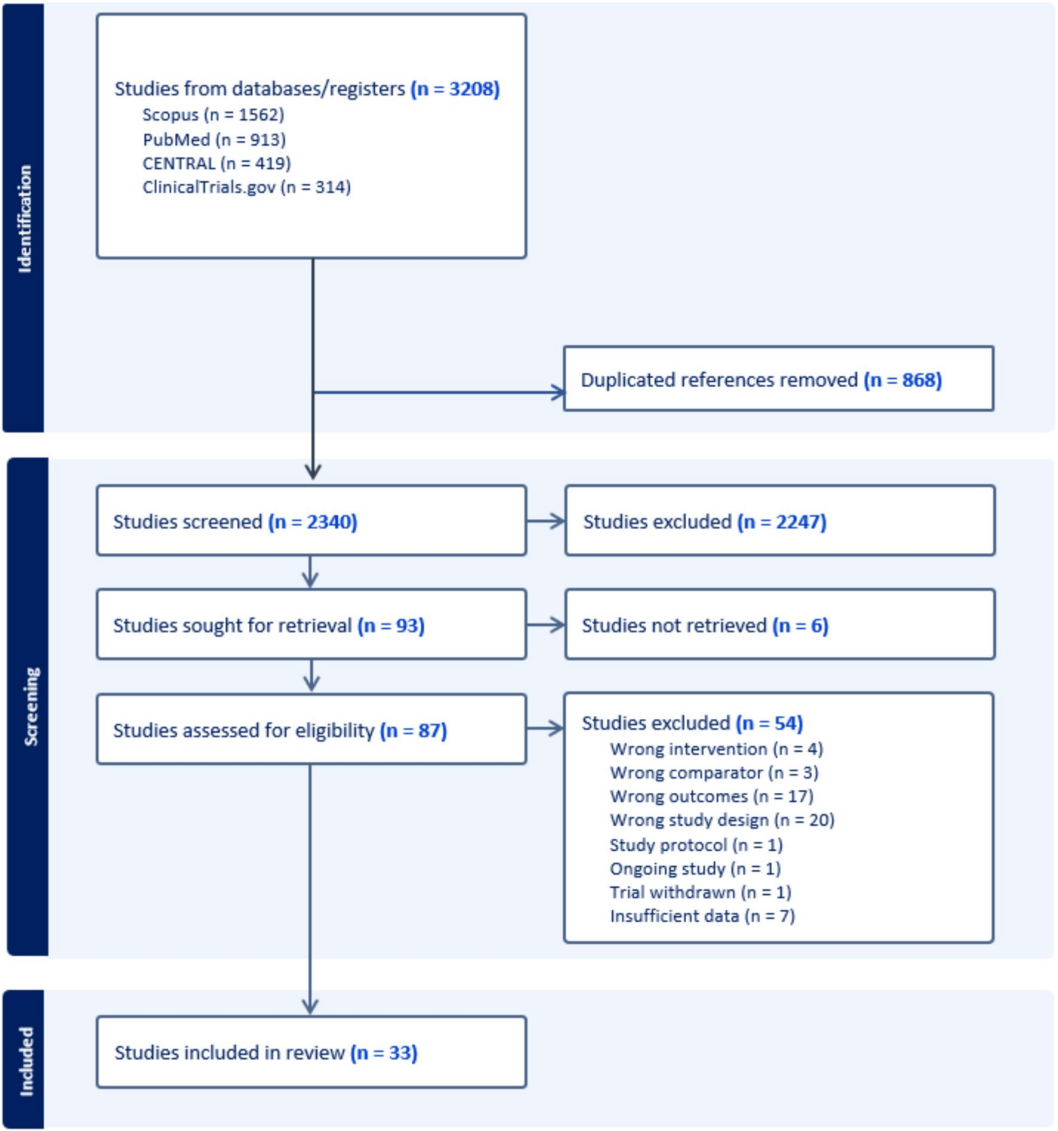
PRISMA flow diagram of selection process

A total of 3208 records were initially identified through database and register searches. After removing 868 duplicates, 2340 studies underwent title and abstract screening, resulting in the exclusion of 2247 records. Of the 93 reports sought for retrieval, 6 could not be obtained. The remaining 87 full-text articles were assessed for eligibility, of which 54 were excluded. Ultimately, 33 studies [20–52] met the inclusion criteria and were included in this systematic review and NMA. The selection process is detailed in Fig. 1.

### Study characteristics

A total of 33 RCTs involving 1118 participants with idiopathic PD were included in this NMA. The studies were conducted across multiple countries, most frequently in Italy, Brazil, Canada, and Thailand. Sample sizes ranged from 7 to 90 participants per study. Most trials (*n* = 28) employed a parallel-group design [20, 22–27, 29–32, 34–40, 42–48, 50–52], while five utilized a crossover design [21, 28, 33, 41, 49]. Disease severity across the included studies spanned Hoehn & Yahr (H&Y) stages 1–4. Although staging was not universally reported, all trials defining inclusion criteria excluded stage 5 patients. Regarding medication status, 22 studies assessed participants in the “ON” state [20–22, 25, 27, 29–32, 34, 36, 37, 39, 40, 42, 45–47, 49–52] and 6 in the “OFF” state [28, 33, 35, 38, 43, 44], while 5 studies did not specify the state [23, 24, 26, 41, 48]. Detailed characteristics of the included studies are summarized in Table 1.

**Table 1:** Characteristics of the included studies

Study	Country	Study design	Stage of PD	ON/OFF assessment	Sample size per treatment arm (total number)	Intervention groups	Intervention details	Outcomes	Follow-up
Almeida 2012 [20]	Canada	RCT, parallel	Not reported H&Y stage	ON state	14/14 (28)	VC vs. C	(1) Overground gait training with transverse lines (2) Overground gait training	Gait velocity	6 weeks
Barbic 2014 [21]	Italy	RCT, crossover (2:1)	H&Y 2–3	ON state	16/8 (16)	SC vs. C	(1) Mechanical pressure stimulation both feet (2) Sham stimulation	Gait velocity	24 h
Braun Janzen 2019 [22]	Canada	RCT, parallel	H&Y 1–2	ON state	11/14/12 (37)	SC + AC vs. AC vs. C	(1) Tapping via contact plate and metallic probe on index finger + RAS (metronome) (2) RAS (metronome) (3) No training	Gait velocity, stride length	Immediate
Bukowska 2016 [23]	Poland	RCT, parallel	H&Y 2–3	N/A	30/25 (55)	AC vs. C	(1) Neurologic music therapy procedure (TIMP, PSE, RAS) (2) Maintained daily life activities	Gait velocity, stride length	4 weeks
Calabrò 2019 [24]	Italy	RCT, parallel	H&Y 2–3	N/A	25/25 (50)	AC VS. C	(1) Treadmill gait training with RAS (2) Treadmill gait training	Gait velocity, stride length	8 weeks
Camerota 2016 [25]	Italy	RCT, parallel	H&Y 2–3	ON state	9/8 (17)	SC VS. C	(1) Repetitive focal muscle vibration (2) Sham stimulation	Gait velocity, stride length	24 h
Carpinella 2017 [26]	Italy	RCT, parallel	H&Y 2–4	N/A	17/20 (37)	VC + AC vs. C	(1) Gamepad with real-time visual and acoustic biofeedback (2) Conventional physiotherapy	Gait velocity	20 sessions
Chaiwanichsiri 2011[27]	Thailand	RCT, parallel	H&Y 2–3	ON state	10/10 (20)	AC vs. C	(1) Treadmill gait training with music (2) Treadmill gait training	Gait velocity, stride length	4 weeks
Chang 2019 [28]	Taiwan	RCT, crossover	Mean (SD) H&Y: 2.14 (0.85)	OFF state	21/21 (21)	AC vs. C	(1) Stepping in place training with metronome (2) Stepping in place training	Gait velocity, stride length	Immediate
Cui 2017 [29]	China	RCT, parallel	H&Y 3	ON state	20/20 (40)	VC + AC vs.C	(1) Combined visual cue (straight line) and auditory cue (metronome) with physiotherapy (2) Conventional physiotherapy	Gait velocity	4 weeks
Cursino 2018 [30]	Brazil	RCT, parallel	H&Y 1–3	ON state	7/7 (14)	AC vs. C	(1) Treadmill gait training with metronome (2) Treadmill gait training	Gait velocity	6 weeks
de Bruin 2010 [31]	Canada	RCT, parallel	H&Y 2–3	ON state	11/11 (22)	AC vs. C	(1) Walk with music playlist (2) Regular activities	Gait velocity, stride length	13 weeks
De Icco 2015 [32]	Italy	RCT, parallel	H&Y 2–4	ON state	11/11/24 (46)	VC vs. AC vs. C	(1) Gait training with colored stripes (2) Gait training with metronome (3) Overground gait training	Gait velocity, stride length	4 weeks
De Pandis 2025 [33]	Italy	RCT, crossover	H&Y ≥ 2	OFF state	74/74 (74)	SC vs. C	(1) AMPS (2) Sham AMPS	Gait velocity, stride length	3 weeks
El-Tamawy 2012 [34]	Egypt	RCT, parallel	H&Y 2–3	ON state	15/15 (30)	SC vs. C	(1) Treadmill training with vibratory stimuli (2) Routine physiotherapy	Gait velocity, stride length	8 weeks
Galli 2018 [35]	Italy	RCT, parallel	Not reported H&Y stage	OFF state	14/14 (28)	SC vs. C	(1) AMPS (2) Sham AMPS	Gait velocity, stride length	6 sessions
Ginis 2016 [36]	Belgium, Isarael	RCT, parallel	H&Y 2–3	ON state	22/18 (40)	AC vs. C	(1) Walk with smartphone application provided verbal biofeedback (2) Gait advice	Gait velocity, stride length	6 weeks
Harro 2014 [37]	USA	RCT, parallel	H&Y 1–3	ON state	10/10 (20)	AC vs. C	(1) Overground gait training with RAC (2) Speed-dependent treadmill training	Gait velocity	6 weeks
Kleiner 2018 [38]	Brazil	RCT, parallel	H&Y stage 1–4	OFF state	15/15 (30)	SC vs. C	(1) AMPS (2) Sham AMPS	Gait velocity	8 sessions
Laswell 2015 [39]	Canada	RCT, parallel	Not reported H&Y stage	ON state	13/13 (26)	SC vs. C	(1) Treadmill training with somatosensory feedback (ankle bracelet) (2) Treadmill training	Gait velocity, stride length	6 weeks
Lirani-Silva 2017 [40]	Brazil	RCT, parallel	H&Y stage 1–3	ON state	10/9 (19)	SC vs. C	(1) Texture insoles (2) Conventional insoles	Gait velocity, stride length	1 week
Marques 2022 [41]	Brazil	RCT, crossover	H&Y stage 2–4	N/A	28/28 (28)	SC vs. C	(1) AMPS (2) Sham AMPS	Gait velocity	Immediate
Miyahara 2024 [42]	Thailand	RCT, parallel	H&Y 1–4	ON state	30/30/30 (90)	VC vs. SC vs. C	(1) Laser cane (2) Therapeutic Thai acupressure over plantar of feet (3) Sham control: light touch over plantar of feet	Gait velocity, stride length	Immediate
Pagnussat 2018 [43]	Brazil	RCT, parallel	H&Y stage 1–4	OFF state	16/16 (32)	SC vs. C	(1) AMPS (2) Sham AMPS	Gait velocity, stride length	4 weeks
Pagnussat 2020 [44]	Brazil	RCT, parallel	H&Y stage 1–4	OFF state	10/7 (17)	SC vs. C	(1) AMPS (2) Sham AMPS	Gait velocity	4 weeks
Phuenpathom 2022 [46]	Thailand	RCT, parallel	H&Y stage 1–4	ON state	15/15 (30)	SC vs. C	(1) Combined vibratory device at Achilles tendons and silicone pad (2) No stimulation	Stride length	Immediate
Phuenpathom 2023 [45]	Thailand	RCT, parallel	H&Y stage 1–4	ON state	20/20 (40)	SC vs. C	(1) Shoes embedded with vibratory stimulation at Achilles tendons and soft thickened silicon pad (2) Sham shoes	Stride length	Immediate
Pollet 2023 [47]	Italy	RCT, parallel	Not reported H&Y stage	ON state	21/21 (42)	SC vs. C	(1) Custom-made insoles (2) Sham insoles	Gait velocity, stride length	10 weeks
Schlick 2016 [48]	Germany	RCT, parallel	H&Y stage 2–4	N/A	10/10 (20)	VC vs. C	(1) Treadmill walking with footprint projection (2) Treadmill training	Gait velocity, stride length	6 weeks
Serrao 2019 [49]	Italy	RCT, crossover	H&Y stage 1–4	ON state	40/40 (40)	VC vs. C	(1) Visual cue training (parallel transverse lines on floor) + progressive modular rebalancing technique (2) Conventional physiotherapy	Gait velocity	8 weeks
Shen 2012 [50]	Hongkong	RCT, parallel	H&Y stage 1–4	ON state	14/14 (28)	VC vs. C	(1) Repetitive step training with preparatory visual cues (computer screen) (2) Lower limb strengthening training	Gait velocity, stride length	4 weeks
Shen 2014 [51]	Hongkong	RCT, parallel	H&Y stage 2–3	ON state	22/23 (45)	VC vs. C	(1) Repetitive step training with preparatory visual cues (computer screen) (2) Lower limb strengthening training	Gait velocity, stride length	3 months
Tinuan 2024 [52]	Thailand	RCT, parallel	H&Y stage 2–3	ON state	18/18 (36)	VC vs. C	(1) Multi-visual-cue mat (color contrasts, small squares, numbers) (2) Regular daily activities	Gait velocity, stride length	3 months

*AC* auditory cue, *AMPS* automated mechanical peripheral stimulation, *C* control, *H&Y* Hoehn and Yahr, *N/A* not applicable, *PSE* patterned sensory enhancement, *RAC* rhythmic auditory-cued, *RAS* rhythmic auditory stimulation, *RCT* randomized controlled trial, *SC* somatosensory cue, *TIMP* therapeutic instrumental music performance, *VC* visual cue

Interventions were categorized into four primary cueing modalities: visual, auditory, somatosensory, and combined cues. VC interventions included transverse floor lines, colored stripes, laser canes, footprint projections, and computer-screen displays [20, 32, 42, 48–52]. AC primarily involved Rhythmic Auditory Stimulation (RAS), metronomes, music playlists, and verbal biofeedback [22–24, 27, 28, 30–32, 36, 37]. SC interventions encompassed automated mechanical peripheral stimulation (AMPS), vibratory stimulation, textured insoles, and tactile feedback devices [21, 25, 33–35, 38–47], while combined cues integrated multiple modalities (e.g., VC + AC or SC + AC) [22, 26, 29]. Control groups (C) received conventional physiotherapy, sham stimulation, or engaged in regular daily activities.

Gait velocity was reported in 31 studies [20–44, 47–52], 23 of which also reported stride length [22–25, 27, 28, 31–36, 39, 40, 42, 43, 45–48, 50–52]. Follow-up periods varied considerably, ranging from immediate post-intervention assessments to 3 months; however, most studies (*n* = 16) conducted follow-up assessments at 4–8 weeks post-intervention.

### Risk of bias assessment

**Fig. 2:**
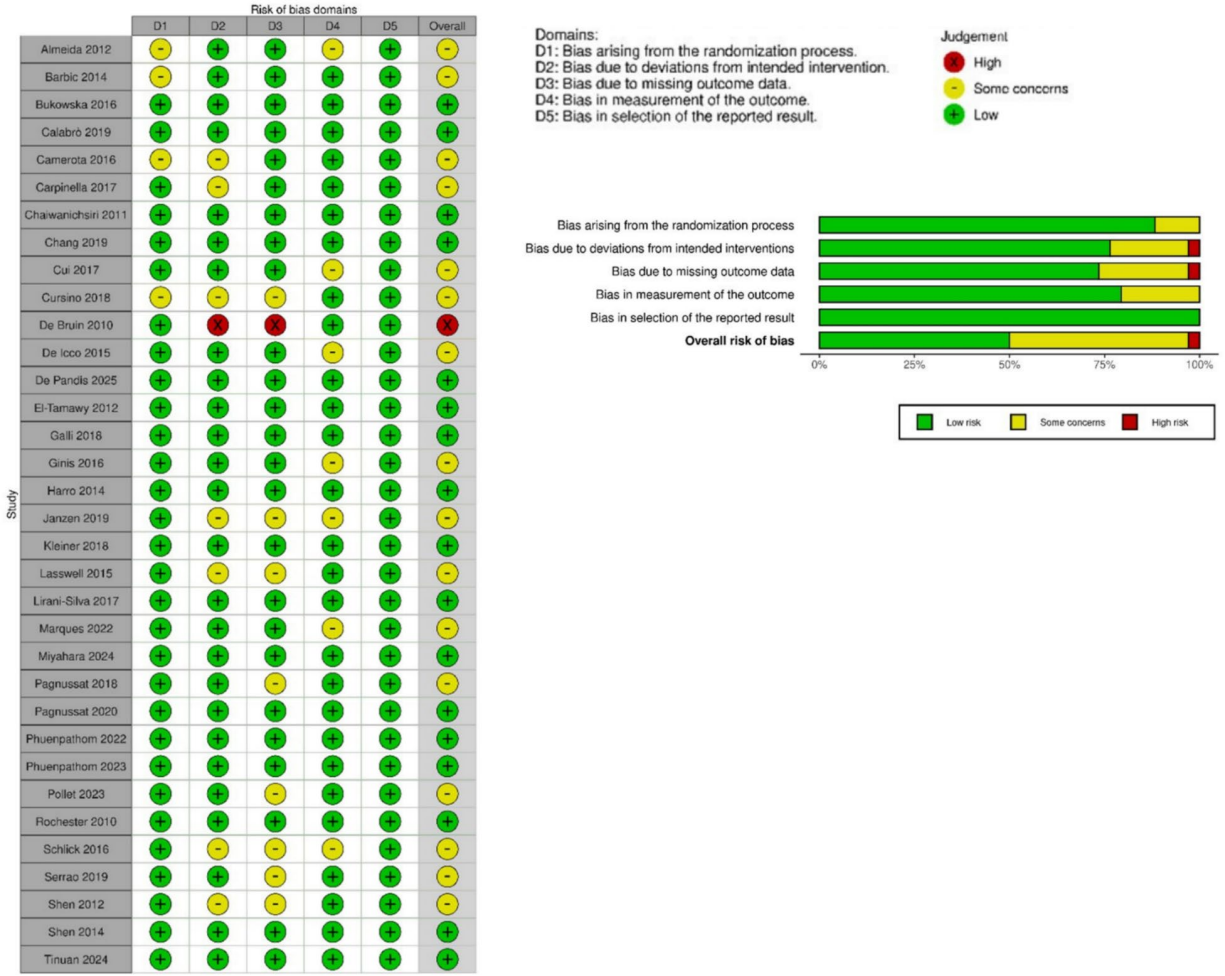
Summary table and graph of risk of bias assessment

Methodological quality was evaluated using the RoB 2 tool. Overall, 16 studies (48.5%) were categorized as low risk [23, 24, 27, 28, 33–35, 37, 38, 40, 42, 44–46, 51, 52], 16 (48.5%) as having some concerns [20–22, 25, 26, 29, 30, 32, 36, 39, 41, 43, 47–50], and one (3%) as high risk [31]. The primary driver of bias was the missing outcome data domain, where eight studies [22, 30, 39, 43, 47–50] raised some concerns and one [31] was rated as high risk due to significant attrition. This was followed by bias arising from deviations from intended interventions and bias in the measurement of the outcome, both of which contributed to the ‘some concerns’ classifications. A summary of these assessments is provided in Fig. 2.

### Network geometry and result synthesis

#### Gait velocity

**Fig. 3:**
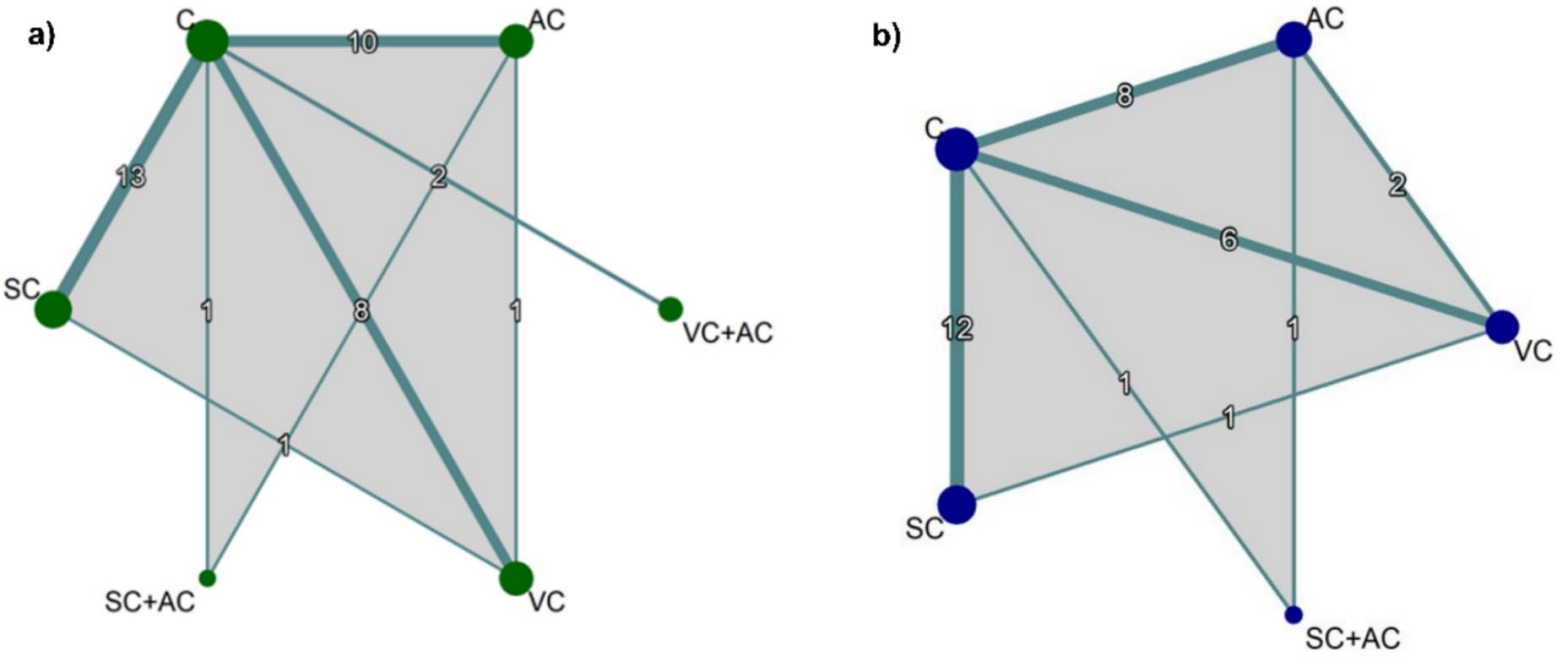
Network geometry of eligible comparisons for **a** gait velocity, and **b** stride length. Nodes represent specific interventions, with node size proportional to the total number of randomized participants. Lines indicate direct head-to-head comparisons, with line thickness corresponding to the number of studies available for each comparison

The network geometry for gait velocity included 31 studies [20–44, 47–52] and is presented in the network plot (Fig. 3a). The network was well connected with the control group (C) as the most common comparator (*n* = 580 participants). Direct comparisons were most frequent between SC and C (13 trials) [21, 25, 33–35, 38–44, 47] and AC and C (10 trials) [22–24, 27, 28, 30–32, 36, 37].

**Fig. 4:**
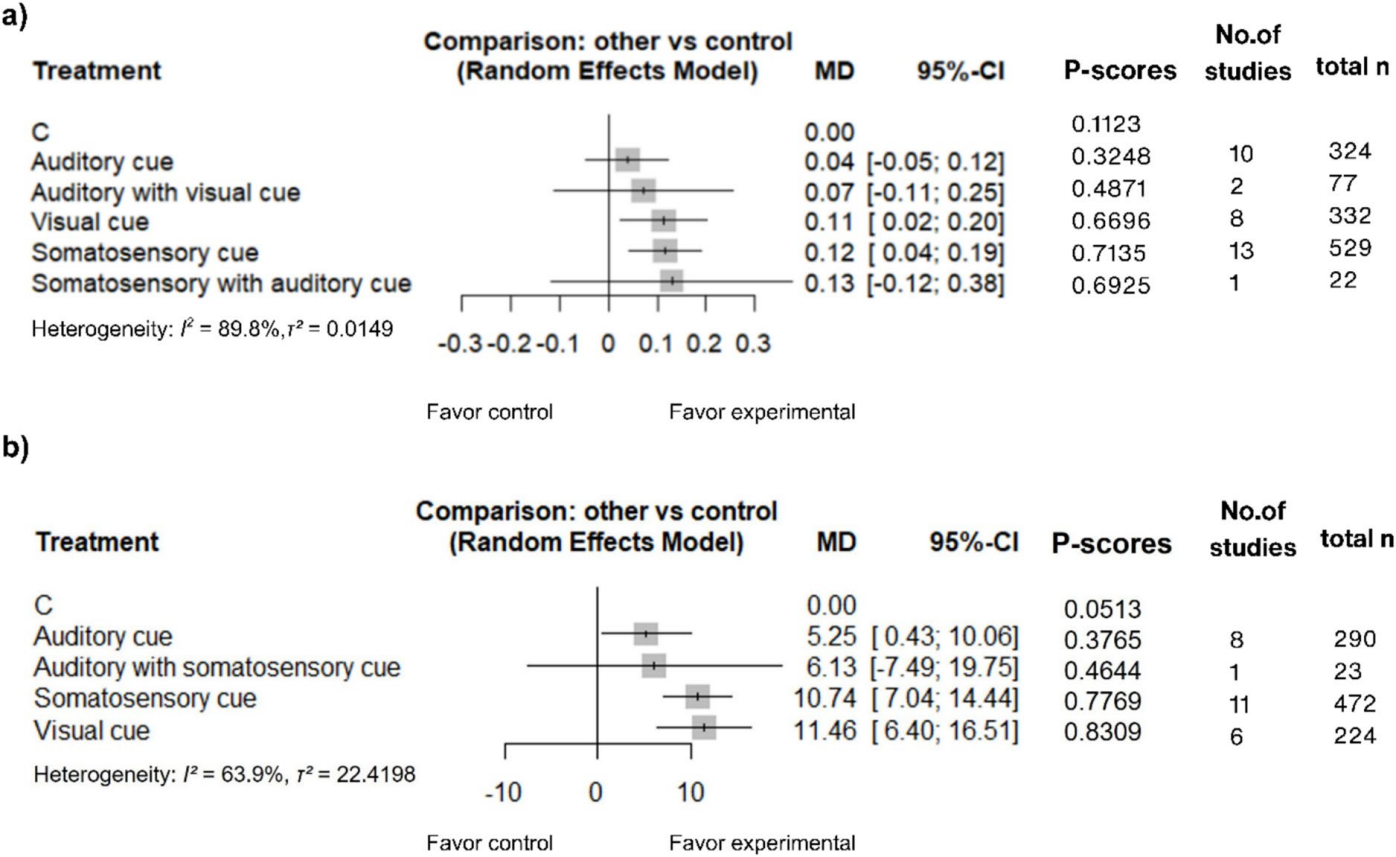
Forest plots and treatment rankings for **a** gait velocity, and **b** stride length. The plots display the comparative efficacy of each cueing intervention versus the control group. Interventions are ranked according to their *P*-scores, representing the probability of each strategy being the most effective treatment

**Fig. 5:**
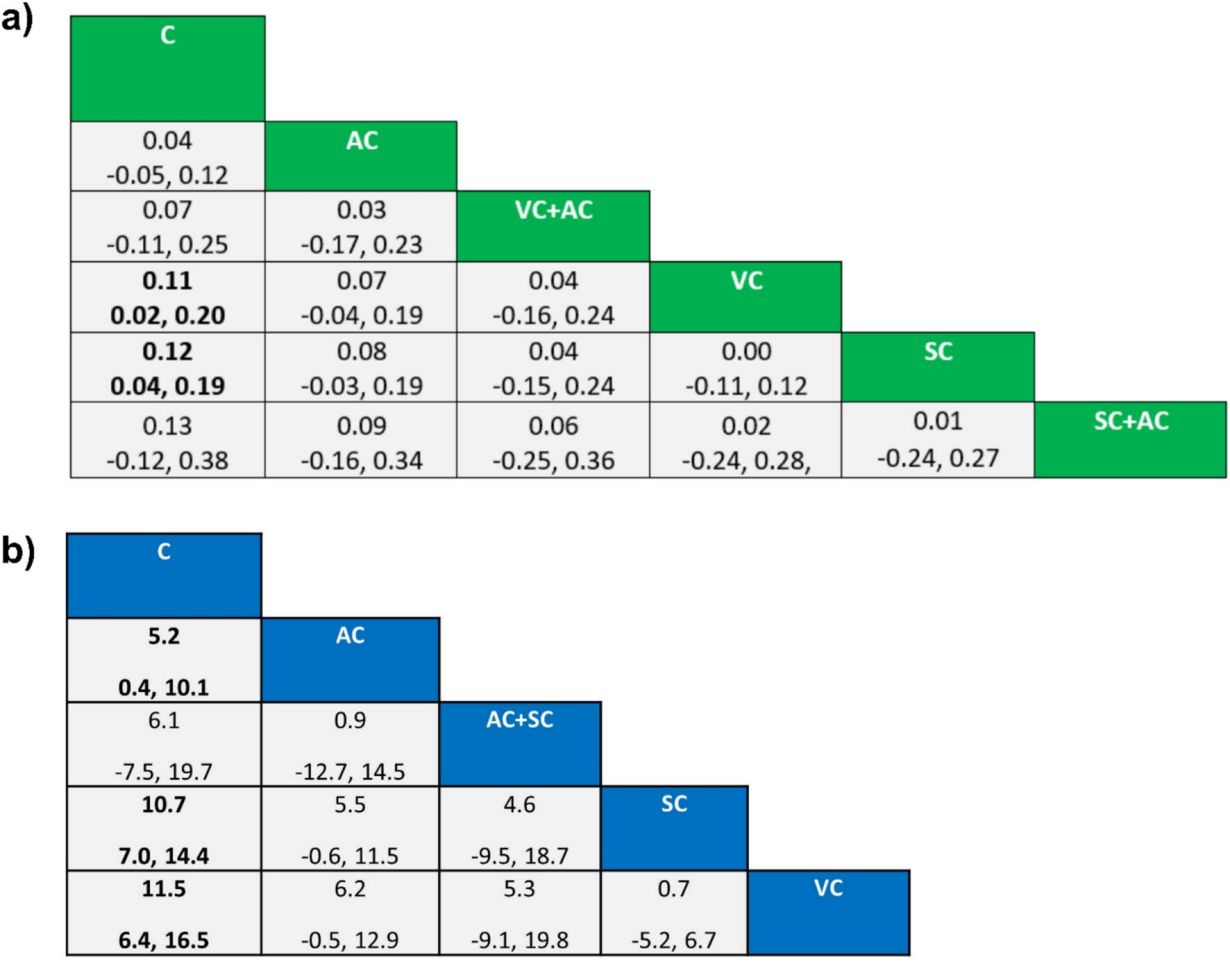
League table of comparative efficacy for **a** gait velocity and **b** stride length. Estimates are presented as mean differences (MD) with 95% confidence intervals. For each cell, the MD represents the comparison of the column-defining intervention against the row-defining intervention; a positive MD value indicates that the row-defining treatment is superior. Results that reached statistical significance are highlighted in bold

The results of the NMA and direct pairwise comparisons are shown in the forest plots (Figs. 4a and S1; See supplementary file 2) and league table (Fig. 5a). In the NMA, SC significantly improved gait velocity when compared with control (MD = 0.12 m/s, 95% CI 0.04–0.19). VC also demonstrated a significant benefit over control (MD = 0.11 m/s, 95% CI 0.02 to 0.20). Notably, the corresponding direct pairwise comparison of VC versus C did not reach statistical significance in the random-effects model (MD = 0.12 m/s, 95% CI − 0.04 to 0.27), highlighting the increased precision gained through the incorporation of indirect evidence (Fig. S1a). AC showed a small, nonsignificant improvement in gait velocity in the NMA (MD = 0.04 m/s, 95% CI − 0.05 to 0.12). The combination of SC + AC yielded the highest point estimate for improvement (MD = 0.13 m/s), although it did not reach statistical significance (95% CI − 0.12 to 0.38). Similarly, VC + AC showed a nonsignificant improvement (MD = 0.07 m/s, 95% CI − 0.11 to 0.25). No significant differences were observed in head-to-head comparisons between any active intervention modalities.

According to *P*-scores (Fig. 4a), SC was ranked as the most effective intervention for improving gait velocity (*P*-score = 0.7135), followed closely by SC + AC (*P*-score = 0.6925), and VC (*P*-score = 0.6696). The control group ranked lowest (*P*-score = 0.1123).

Substantial global heterogeneity was observed across the network model (*I*^*2*^ = 89.9%, *τ*^2^ = 0.0149). The global assessment of inconsistency was also statistically significant (*Q* = 285.43, *d.f.* = 29, *P* < 0.001), indicating potential evidence conflicts across the network. Furthermore, significant heterogeneity within specific study designs was present (*Q* = 267.35, *d.f.* = 24, *P* < 0.001). Collectively, these findings suggest that the relative efficacy of the external sensory cueing interventions fluctuated substantially across different trial settings, patient cohorts, or study methodologies. Despite these findings, node-splitting analysis revealed no significant inconsistency between direct and indirect evidence for any specific comparisons (*P* > 0.05 for all), supporting the general coherence of the network.

To assess the impact of data transformations, a sensitivity analysis was conducted excluding the single small-scale study that required converting medians and quantiles to means [25]. The results for gait velocity remained robust, with no significant changes in effect sizes or modality rankings, confirming that the imputed statistics did not bias the overall pooled estimates (Fig. S3). Owing to the nature of the available literature, sensitivity analyses excluding high-risk or small-sample studies were not feasible; specifically, only a single study [31] was categorized as high risk, and 29 of the 33 included trials featured sample sizes fewer than 50 participants.

**Fig. 6:**
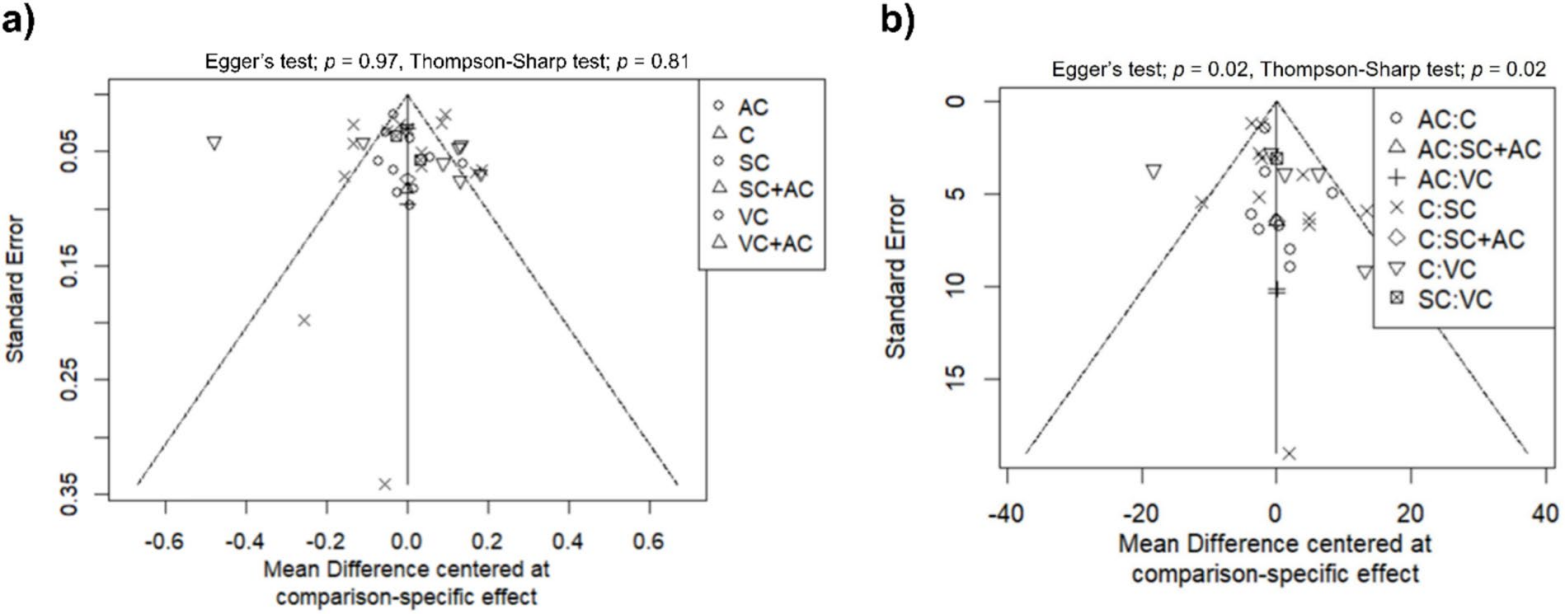
Funnel plots assessing publication bias for **a** gait velocity, and **b** stride length

Visual inspection of the funnel plot (Fig. 6a) showed a generally symmetrical distribution of studies, despite several outliers. Formal statistical testing confirmed a lack of significant publication bias (Egger’s test: *P* = 0.97; Thompson–Sharp test: *P* = 0.81).

#### Stride length

The network architecture for stride length, encompassing 23 studies [22–25, 27, 28, 31–36, 39, 40, 42, 43, 45–48, 50–52], is presented in the network plot (Fig. 3b). The network exhibited robust connectivity, with the control group (C) serving as the primary common comparator (*n* = 460 participants). No studies evaluated the combined SC + AC intervention for this outcome. Consequently, the network comprised four active interventions: VC, AC, SC, and VC + AC.

The comparative efficacy results from the NMA and direct pairwise assessments are detailed in the forest plots (Figs. 4b and S2) and the league table (Fig. 5b). In the NMA, VC, SC, and AC all demonstrated statistically significant improvements in stride length compared to control, with MD of 11.46 cm, 10.74 cm, and 5.25 cm, respectively. Conversely, the combined AC + SC intervention did not reach statistical significance when compared to control (MD = 6.13 cm, 95% CI − 7.49 to 19.75). No statistically significant differences were observed in head-to-head comparisons between any of the active intervention modalities.

Treatment hierarchies derived from *P*-scores (Fig. 4b) indicated that VC had the highest probability of being the most effective strategy for increasing stride length (*P*-score = 0.8309). This was followed by SC (*P*-score = 0.7769), AC + SC (*P*-score = 0.4644), and AC (*P*-score = 0.3765). Consistent with the gait velocity findings, the control group was identified as the least effective option (*P*-score = 0.0513).

The network model exhibited substantial global heterogeneity (*I*^2^ = 63.9%, *τ*^2^ = 22.42). A global assessment of inconsistency was statistically significant (*Q* = 66.53, *d.f.* = 24, *P* < 0.001), indicating discrepancies in treatment effects across the network. Furthermore, the test of heterogeneity within designs reached significance (*Q* = 51.88, *d.f.* = 18, *P* < 0.001), reflecting substantial variability among the included trials. Crucially, node-splitting analysis identified significant inconsistency between the direct and indirect evidence specifically for the comparisons of AC versus VC (*P* = 0.0048) and AC versus C (*P* = 0.0080).

To investigate the sources of this variability, sensitivity analyses were performed by systematically excluding specific modalities. The exclusion of AC failed to reduce the model’s instability (*I*^2^ = 68.9%, Fig. S4a). In contrast, removing VC from the network resulted in a marked reduction in heterogeneity (*I*^2^ = 37.3%, Fig. S4b). These findings suggest that the variability associated with the VC studies [32, 42, 48, 50–52] significantly influenced the overall consistency and heterogeneity of the network analysis. A robustness check was performed by omitting the individual small-sample trial that necessitated the conversion of median and quantile values into mean and standard deviation [25]. The findings for stride length remained stable, with no meaningful shifts in effect sizes (Fig. S4c). Consistent with the gait velocity outcome, sensitivity analyses based on risk of bias and small sample size study were not conducted due to the aforementioned data constraints.

Regarding publication bias, visual inspection of the funnel plot (Fig. 6b) showed a general symmetrical distribution. However, formal statistical tests reached statistical significance (Egger’s test: *P* = 0.02; Thompson–Sharp test: *P* = 0.02). These findings suggest that the observed deviations are likely attributable to small-study effects rather than true publication bias. A sensitivity analysis based on the sample size was considered unfeasible, as only four studies featured a sample size of *n* ≥ 50 [23, 24, 33, 53].

### Post hoc synthesis of FOG outcome

Beyond spatiotemporal parameters, we examined the impact of cueing on FOG, a critical symptom that often resists dopaminergic therapy. The data on FOG were limited and characterized by diverse assessment methodologies, including subjective questionnaires (FOG-Q, NFOG-Q) and objective kinetic measurements, such as the number of episodes, duration, and percentage of FOG [26, 29, 33, 36, 42, 45, 46, 48, 52].

Regarding SC, the recent trials suggest strong efficacy, particularly when utilizing multimodal stimulation such as combined vibratory and pressure cues. These interventions significantly reduced the percentage, frequency, and duration of freezing episodes as compared to sham control [45, 46]. In addition, delivering SC via therapeutic Thai acupuncture was found to reduce the number of freezing episodes [42]. However, alternative SC protocols, such as AMPS, yielded nonsignificant results in certain crossover designs [33].

Evidence for VC remains inconsistent across the literature. Certain technologies, such as laser-light canes [42] or multi-visual cue step training with targeted environmental markers [52], demonstrated significant improvements in FOG steps and FOG-Q scores, respectively [42, 52]. Conversely, another study reported no significant benefit when adding VC to treadmill training compared to treadmill training alone.

Finally, standalone AC showed limited efficacy, with reported outcomes failing to reach statistical significance on the NFOG-Q [36]. Combined VC and AC interventions produced highly variable results, ranging from significant clinical improvements in FOG-Q scores to no observable effect [26, 29].

## Discussion

This network meta-analysis establishes an evidence-based hierarchy for external sensory cueing in PD, advocating for a shift from generic protocols toward a phenotype-targeted approach. For the primary objective of increasing gait velocity, our results indicate that SC and VC offer comparable efficacy (*P*-scores of 0.71 and 0.67, respectively), with both modalities demonstrating a superior capacity to augment speed compared to other interventions. A similar trend was observed for stride length, where VC and SC again emerged as the top-ranked modalities (*P*-scores of 0.83 and 0.78, respectively). Notably, while AC is the most widely accessible and easily integrated into everyday life, our findings demonstrate that it is significantly less effective than SC or VC for specifically augmenting these two spatiotemporal parameters.

Clinically, the effectiveness of VC in increasing gait velocity and stride length likely stems from its ability to bypass deficient internally generated motor signals. By providing external spatial references, VC engages the dorsal visual stream, involving the posterior parietal and lateral premotor cortices, allowing patients to scale their movements based on environmental landmarks [54]. This mechanism specifically addresses the reduced amplitude characteristic of PD gait; by increasing stride length through enhanced sensorimotor integration in the motor and visual cortices, VC subsequently drives significant improvements in overall gait velocity [7, 55]. Our findings align with the previous studies demonstrating the effectiveness of VC in increasing stride length and reducing stride length variability [4, 55].

Excluding VC studies significantly reduced network heterogeneity for stride length from 63.9 to 37.3%, suggesting that VC interventions introduced substantial variability into the model. This inconsistency likely arises from the marked diversity in delivery methods, which ranged from static environmental markers, such as floor tape, to sophisticated wearables like laser-light canes and head-mounted displays [32, 42, 48, 50–52], or dynamic foot projection on treadmill [48]. Although fixed markers provide a stable “target” for overstepping in controlled settings, dynamic wearable cues may elicit varying kinematic responses contingent upon a participant’s baseline deficits and device familiarity. Ultimately, this technological breadth and the lack of standardized protocols (e.g., overground versus treadmill walking) explain why VC interventions demonstrated less consistency than the more uniform rhythmic auditory or somatosensory stimulations. Specifically, when look into direct head-to-head comparison, the use of laser-light canes and multi-visual cue step training demonstrated significant improvements in gait velocity compared to controls. Furthermore, a superior effect on stride length was observed with multi-visual cue mats, laser-light canes, and overground training with white lines. Beyond statistical significance, these findings highlight a critical distinction in clinical application. Although white lines and cue mats are effective for intensive training sessions in hospital or research settings, they are less adaptable to the complex, changing environments of daily life. In contrast, the laser-light cane represents a highly portable and “real-world” adaptable solution, providing a consistent spatial target regardless of the setting.

SC aims to restore the proprioceptive feedback system and “sensorimotor gating” mechanisms that are frequently dysfunctional in PD [56]. The current interventions often combine SC during walking tasks with multiple sessions of sensory stimulation to restore proprioceptive function and optimize motor output [25, 33, 35, 38, 41, 43, 44].Our direct head-to-head analysis reveals that the efficacy of SC is highly dependent on the delivery method. Specifically, traditional Thai acupuncture and the majority of studies utilizing AMPS demonstrated significant improvements in both gait velocity and stride length [33, 38, 43, 44]. Furthermore, combined vibration and pressure stimulation applied to the feet during gait proved effective for both outcomes [45, 46]. In contrast, wearable devices providing vibratory stimulation during walking yielded inconsistent results [34, 39], while vibration applied to the lower limbs during nongait tasks [21, 25] or the use of tactile insoles failed to improve velocity or stride length [40, 47].

While AMPS and multimodal vibratory-pressure systems offer robust therapeutic benefits, their availability is currently often limited to specialized clinical or research settings. Conversely, Thai acupuncture—where accessible—provides a more immediate nontechnological alternative. For real-world management, a significant challenge remains in translating the success of multimodal stimulations, such as combined pressure and vibration, into mass-produced wearable products that are affordable for the general patient population.

AC mitigates gait impairments in PD by bypassing dysfunctional neural circuits. By substituting deficient pallidocortical projections and activating the supplementary motor area, rhythmic stimuli facilitate motor initiation and stabilize spatiotemporal parameters [8, 57]. In contrast to visual or somatosensory cues, auditory cueing is more readily accessible through the use of metronomes, rhythmic auditory stimulation, or music. These can be easily integrated into both overground and treadmill-based walking training, as well as everyday life. However, our findings indicate that while AC effectively increased stride length, it failed to significantly improve gait velocity as a standalone intervention. This discrepancy may stem from the AC frequency; evidence suggests that AC rates must be individualized to address specific gait abnormalities [58, 59]. Currently, there is no consensus on the optimal setting, with therapeutic rates varying from below to significantly above a patient’s baseline cadence [4]. AC frequencies set above baseline cadence typically improve gait velocity, while lower frequencies are associated with increased stride length [58, 59]. On-demand cueing or open-loop cueing are increasingly being explored as tailored approaches to enhance spatiotemporal gait parameters [60, 61]. Moreover, despite being frequently overshadowed by motor and cognitive symptoms, hearing loss affects over 70% of individuals with PD, a prevalence significantly higher than in the general elderly population [62]. Research indicates that PD-related auditory dysfunction involves both peripheral sensitivity—marked by elevated high-frequency thresholds—and central auditory nervous system deficits, characterized by reduced cortical auditory evoked potential amplitudes that signal impaired attentional and automatic acoustic processing [63]. These impairments, often linked to noradrenergic and cholinergic depletion, typically manifest asymmetrically and correlate with the side of most severe motor symptoms [64]. Consequently, these deficits may significantly hinder the efficacy of auditory cueing strategies, as the underlying neural mechanisms required to process and respond to rhythmic stimuli are compromised.

To assess clinical impact, we compared our findings to established Minimal Clinically Important Differences (MCID) for PD: 0.082 m/s for gait velocity and 3.6 cm for step length [65]. Our analysis shows that velocity improvements for SC and VC comfortably exceed the MCID, suggesting functionally meaningful gains. The spatial benefits are even more robust. When converted from stride to step length, the increases for VC and SC remain nearly 50% higher than the clinical threshold. In contrast, although AC achieved statistical significance, its estimated effect on step length fell below the MCID. This distinction justifies our ranking: while all three modalities alter gait, SC and VC offer a magnitude of change more likely to result in perceptible improvements in daily walking performance.

Our narrative synthesis indicates that while cueing holds promise for FOG reduction, its efficacy is highly dependent on the modality and delivery method. Specifically, SC and certain VC appear more effective than standalone auditory cueing, aligned with our main findings. The apparent success of somatosensory devices, such as vibratory/pressure stimulation, and spatial visual cues like laser canes may be due to their ability to provide high-saliency feedback that bypasses the impaired basal ganglia-supplementary motor area loops. This is a critical clinical insight, as it suggests these modalities may offer a viable nonpharmacological strategy for patients whose freezing does not respond well to medication. However, the evidence base remains fragmented by small sample sizes and inconsistent outcome measures, ranging from subjective questionnaires to objective kinetic parameters. Given that FOG was not a pre-specified primary endpoint in our original network meta-analysis protocol, these findings should be interpreted with caution. Nevertheless, the positive trends observed in recent SC and VC trials underscore the urgent need for future large-scale studies to prioritize FOG-specific metrics as primary outcomes.

Although our NMA establishes a clear clinical hierarchy with somatosensory and visual cues outperforming standalone auditory stimulation, this ranking should be viewed as an evidence-based framework rather than a rigid protocol. Clinicians should adjust cueing modalities according to patient-specific factors (e.g., sensory impairment or environmental constraints) which may limit their utility. While specialized tools offer robust results for intensive clinical training, portable interventions—such as laser-light canes or wearable vibratory sensors—provide the necessary adaptability for long-term, real-world management. Consequently, external sensory cueing should be integrated into the standard of care for PD as a nonpharmacological treatment that significantly enhances gait quality.

### Limitations of study

Several limitations of the present NMA warrant consideration. First, a primary challenge throughout this synthesis remains the inherent technological heterogeneity across all cueing modalities. As noted in our sensitivity analysis, the high degree of methodological diversity within delivery systems introduced substantial network heterogeneity, potentially affecting the precision of specific effect estimates. In the visual domain, the efficacy of conventional spatial cues may not be directly comparable to advanced digital or wearable-based visual stimuli, as each delivery method engages different levels of optic flow. Similarly, within the auditory domain, the rhythmic precision of a metronome may elicit different neural responses compared to complex music playlists, which involve additional emotional and cognitive processing. This variance is further compounded by the fact that many included trials are small-scale studies, which may be susceptible to “small-study effects” where extreme results are more easily observed in smaller cohorts. Second, while seven theoretical combinations of VC, AC, and SC exist, the available literature only allowed for the evaluation of five strategies. The absence of RCTs investigating VC + SC or triple-modality combinations represents a gap in the evidence base, limiting our ability to determine the absolute hierarchy of synergistic effects.

Third, the included studies exhibited significant clinical heterogeneity regarding disease stage, the presence of FOG, and medication status (e.g., “ON” vs. “OFF” levodopa assessment). Furthermore, the timing of outcome measures varied substantially; while most studies evaluated effects 4–8 weeks post-intervention, others reported only immediate effects, which may confound the assessment of cumulative therapeutic benefits. Finally, while episodic gait abnormalities (FOG) were addressed via a narrative post hoc analysis, they could not be formally included in the meta-analysis due to a scarcity of RCTs reporting uniform, quantifiable FOG outcomes. Establishing how different external sensory triggers influence the frequency and duration of freezing episodes through standardized metrics remains a critical frontier for optimizing gait rehabilitation in PD.

Despite these limitations, the use of a NMA represents a robust, state-of-the-art approach to navigate this complex landscape. By integrating both direct and indirect evidence, the NMA framework allows for a systematic comparison of interventions that have never been tested head-to-head, effectively pooling fragmented data into a cohesive hierarchy of efficacy. Although the high heterogeneity and small trial sizes warrant a cautious interpretation of evidence certainty, this analysis provides the most comprehensive estimation of comparative effectiveness currently available, serving as a vital roadmap for the design of future, standardized large-scale trials.

## Conclusions

Our findings establish a clinical hierarchy for external sensory cueing in PD, demonstrating that SC and VC are the most effective modalities for improving gait performance. Both SC and VC offer comparable and superior efficacy for augmenting gait velocity and stride length, significantly outperforming standalone auditory stimulation. However, given the inherent technological and clinical heterogeneity of the included trials, these rankings should be interpreted with caution. Consequently, technique or device selection should be tailored to the patient’s clinical phenotype and environmental needs to ensure real-world utility and personalized effectiveness.

## Supplementary Information

Below is the link to the electronic supplementary material.

Supplementary file1 (DOCX 14 KB)

Supplementary file2 (DOCX 4729 KB)

## Data Availability

Data sharing is not applicable to this article as no datasets were generated or analysed during the current study.

## Declarations

### Registration

PROSPERO; CRD420251273651 (registered on 15 January 2026).
