# Supplementary material for: External sensory cueing on gait in Parkinson’s disease: a systematic review and network meta-analysis
Source: J Neurol. 2026 May 22;273(6):334. doi: 10.1007/s00415-026-13857-3 (PMC13197277; doi:10.1007/s00415-026-13857-3)
Supplement: Supplementary file 1 — Supplementary file1 (DOCX 14 KB) [file 415_2026_13857_MOESM1_ESM.docx]

**Appendix A.** Search strategies

Search date: August 19, 2025

**Scopus (1562)**

TITLE-ABS-KEY("Parkinson’s disease" OR Parkinsonism) AND TITLE-ABS-KEY("cueing" OR "cueing strategy" OR "external cue*" OR feedback OR "auditory cue*" OR "visual cue*" OR "tactile cue*" OR "rhythmic cue*" OR "sensory cue*" OR "motor cue*" OR metronome OR acoustic OR rhythm* OR music OR laser OR "wearable cueing device" OR insole OR vibration OR touch OR "electrical stimulation" OR  “mechanical stimulation” OR “AMPS”) AND TITLE-ABS-KEY("gait speed" OR "gait velocity" OR locomotion OR "walking performance" OR walking OR ambulation)

**PubMed (913)**

("Parkinson Disease"[MeSH] OR "Parkinson’s disease" OR "Parkinsonism") AND ("Cueing" OR "cueing strategy" OR "external cue*" OR "feedback" OR "auditory cue*" OR "visual cue*" OR "tactile cue*" OR "rhythmic cue*" OR "sensory cue*" OR "motor cue*" OR "metronome" OR "acoustic" OR "rhythm*" OR "music" OR "laser" OR "wearable cueing device" OR "insole" OR "vibration" OR "touch" OR "electrical stimulation" OR “mechanical stimulation” OR “AMPS”) AND ("Gait"[MeSH] OR "gait speed" OR "gait velocity" OR "locomotion" OR "walking performance" OR "walking" OR "ambulation")

**CENTRAL (419)**

("Parkinson’s disease" OR Parkinsonism) AND (cueing OR "cueing strategy" OR (external NEXT cue*) OR feedback OR (auditory NEXT cue*) OR (visual NEXT cue*) OR (tactile NEXT cue*) OR (rhythmic NEXT cue*) OR (sensory NEXT cue*) OR (motor NEXT cue*) OR metronome OR acoustic OR rhythm* OR music OR laser OR "wearable cueing device" OR insole OR vibration OR touch OR "electrical stimulation" OR “mechanical stimulation” OR “AMPS”) AND ("gait speed" OR "gait velocity" OR locomotion OR "walking performance" OR walking OR ambulation)

**ClinicalTrials.gov (314)**

("Parkinson’s disease" OR Parkinsonism) AND (cueing OR "cueing strategy" OR "external cue*" OR feedback OR "auditory cue*" OR "visual cue*" OR "tactile cue*" OR "rhythmic cue*" OR "sensory cue*" OR "motor cue*" OR metronome OR acoustic OR rhythm* OR music OR laser OR "wearable cueing device" OR insole OR vibration OR touch OR  "electrical stimulation" OR "mechanical stimulation" OR AMPS) AND ("gait speed" OR "gait velocity" OR locomotion OR "walking performance" OR walking OR ambulation)
