# Supplementary material for: External sensory cueing on gait in Parkinson’s disease: a systematic review and network meta-analysis
Source: J Neurol. 2026 May 22;273(6):334. doi: 10.1007/s00415-026-13857-3 (PMC13197277; doi:10.1007/s00415-026-13857-3)

**Supplement materials**

**Supplement 1:** Direct head-to-head comparisons of cueing modalities for gait velocity

**Supplement 2:** Direct head-to-head comparisons of cueing modalities for stride length

**Supplement 3:** Sensitivity analyses of cueing modalities for gait velocity

**Supplement 4:** Sensitivity analyses of cueing modalities for stride length

**Supplement 1:** Direct head-to-head comparisons of cueing modalities for gait velocity

Figure S1. Pairwise meta-analysis forest plots for gait velocity. The plots illustrate direct comparisons between specific cueing modalities and controls: (a) visual cues (VC) vs. control (C); (b) auditory cues (AC) vs. control (C); (c) somatosensory cues (SC) vs. control (C); and (d) combined visual and auditory cues (VC+AC) vs. control (C).

Figure S1a: VC vs. C

**
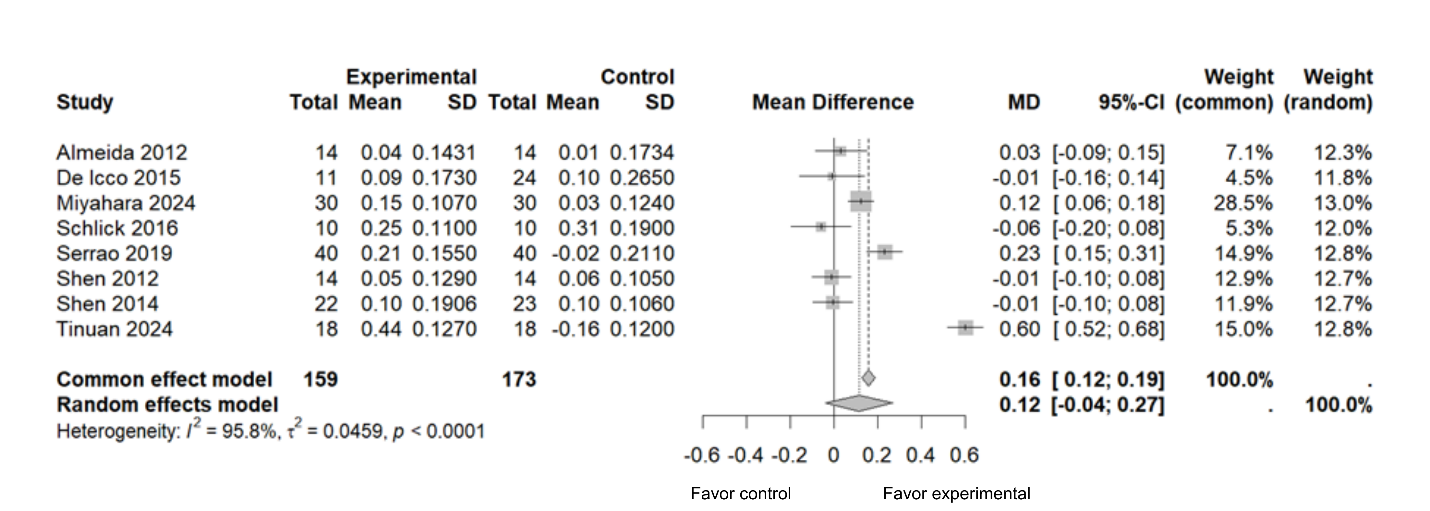
**

Figure S1b: AC vs. C


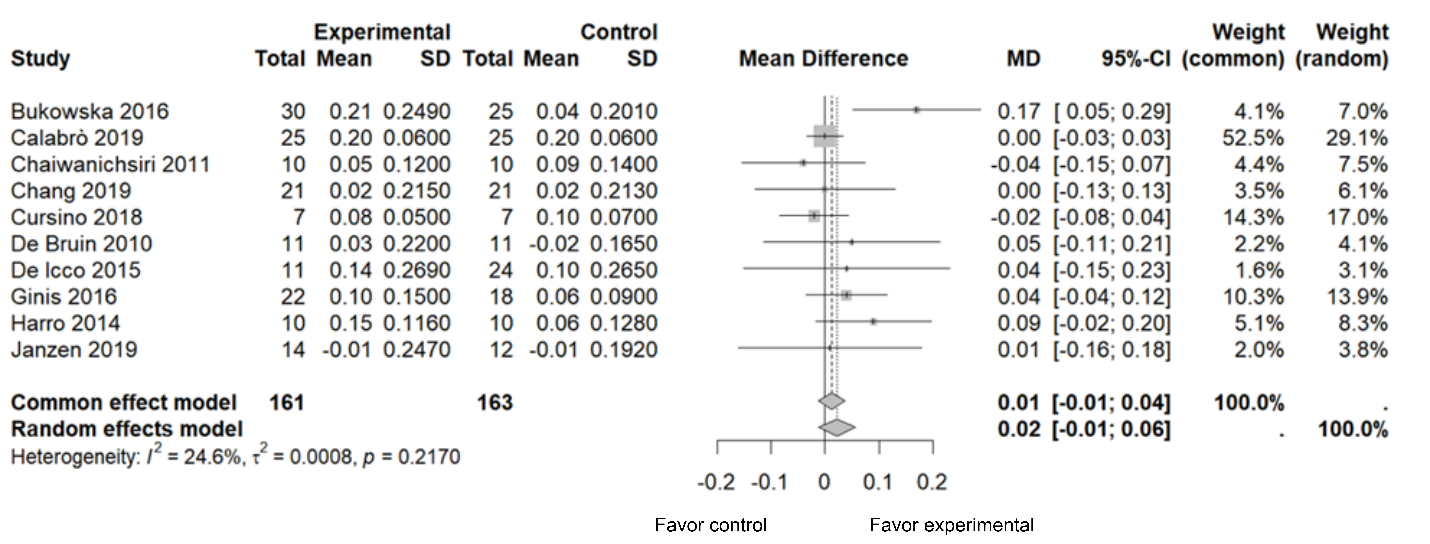


Figure S1c: SC vs. C


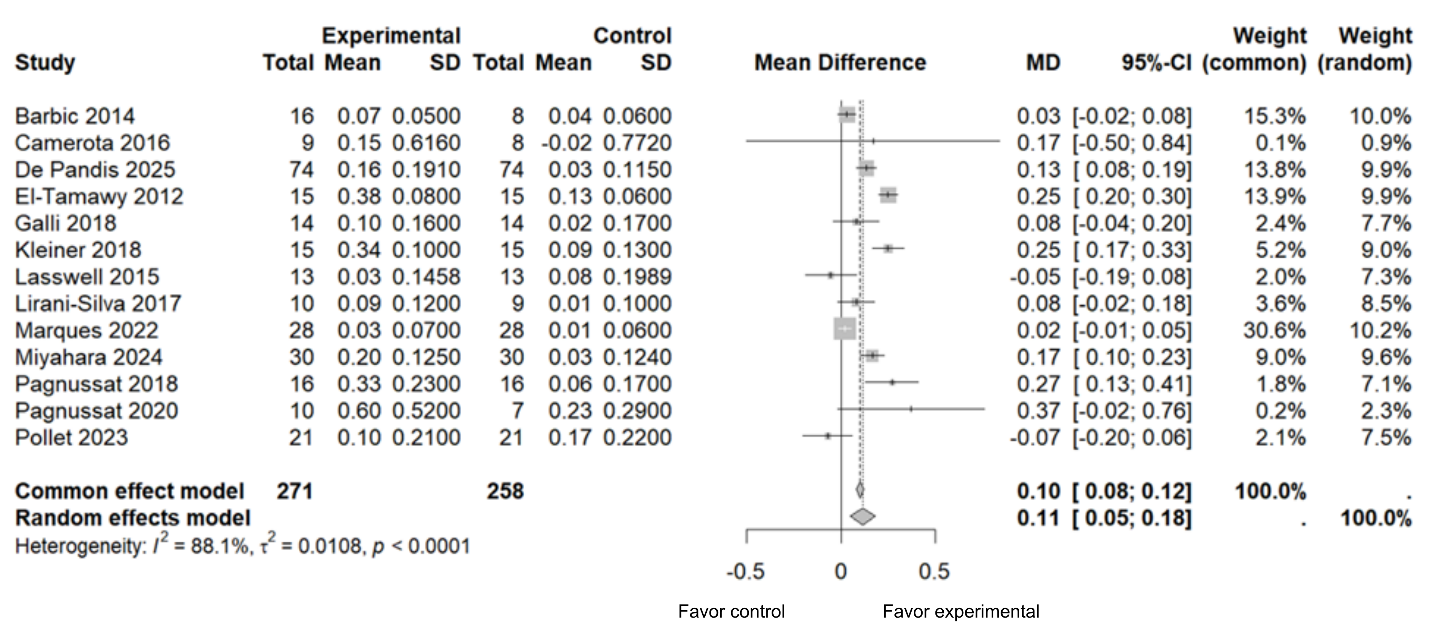


Figure S1d: VC+AC vs. C


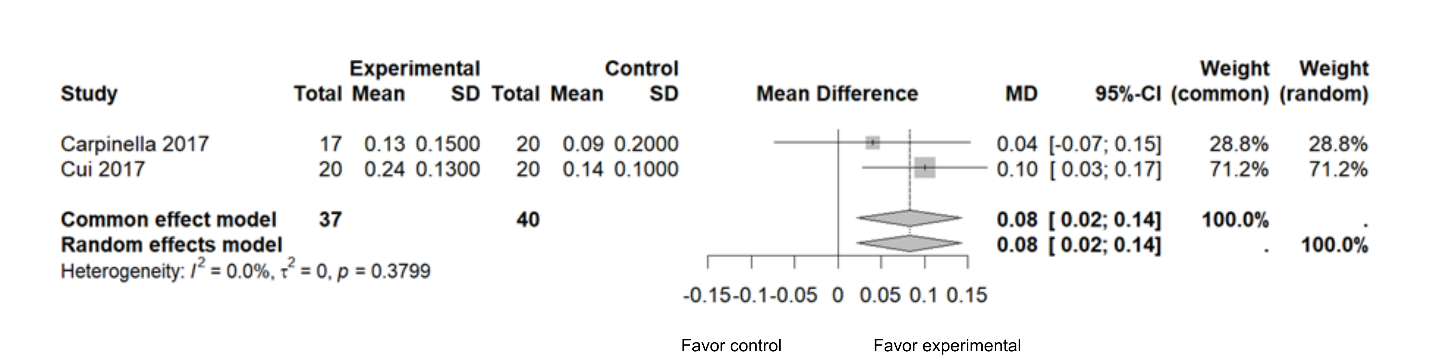


**Supplement 2:** Direct head-to-head comparisons of cueing modalities for stride length

Figure S2. Pairwise meta-analysis forest plots for stride length. The plots present direct evidence for: (a) visual cues (VC) vs. control (C); (b) auditory cues (AC) vs. control (C); (c) somatosensory cues (SC) vs. control (C); and (d) combined auditory and somatosensory cues (AC+SC) vs. control (C).

Figure S2a: VC vs. C


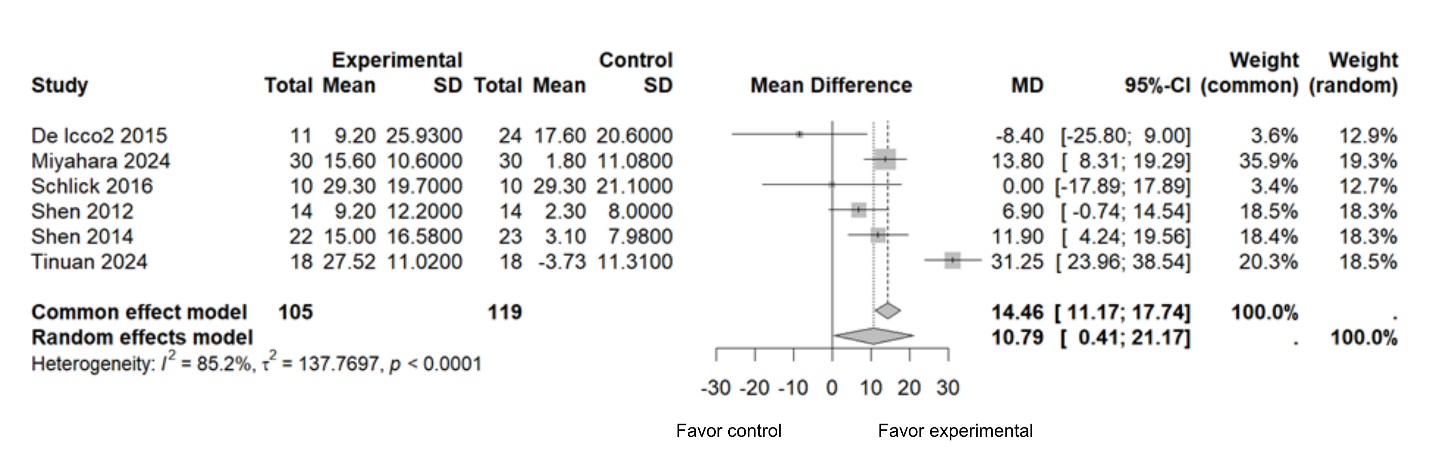


Figure S2b: AC vs. C


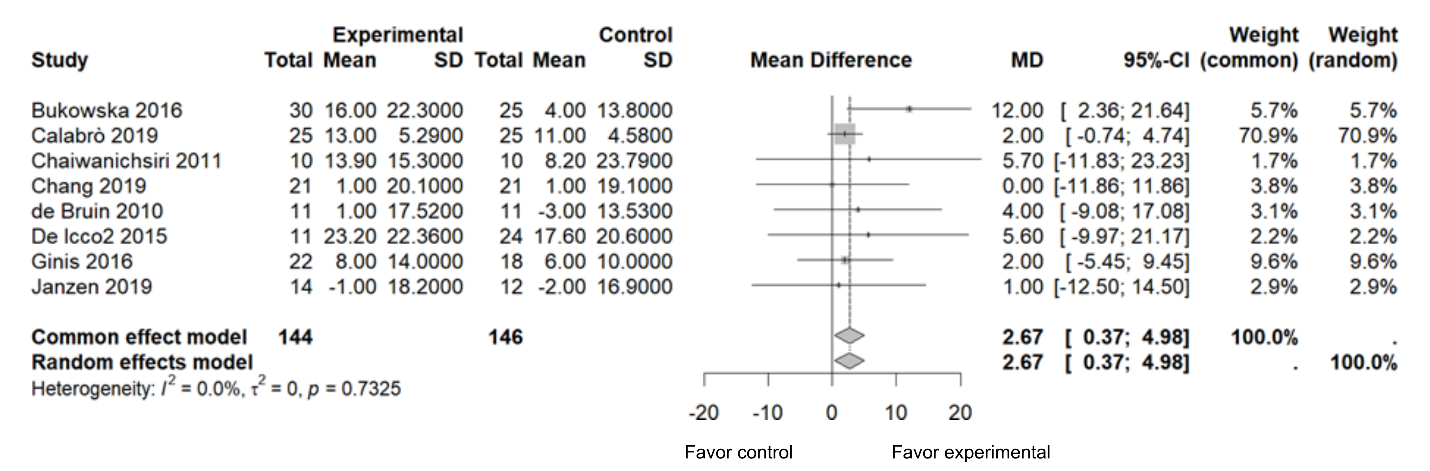


Figure S2c: SC vs. C


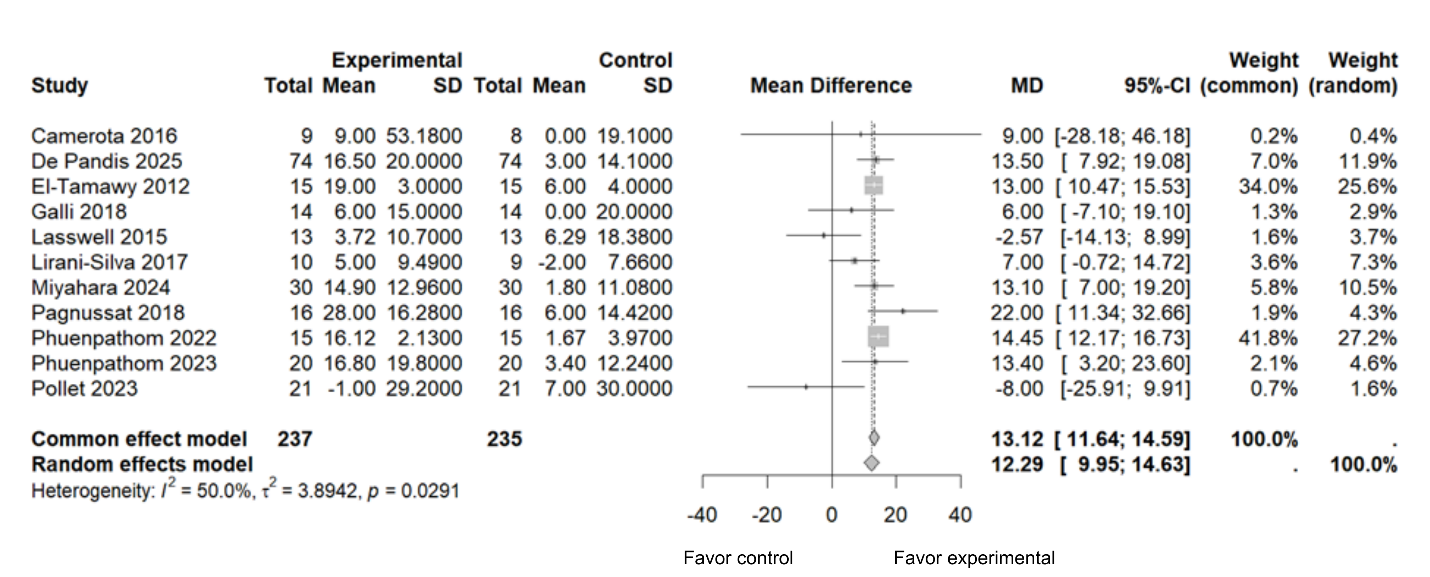


Figure S2d: VC vs. AC


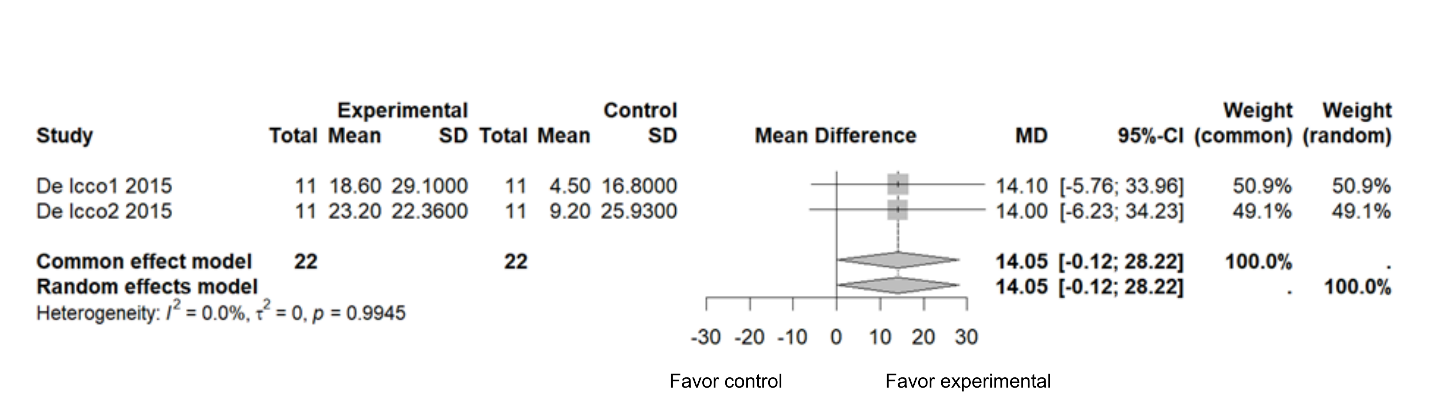


**Supplement 3:** Sensitivity analyses of cueing modalities for gait velocity

Figure S3. Sensitivity analysis forest plots for gait velocity. This plot evaluates the impact of data transformation (median to mean) by displaying the comparative efficacy of interventions versus control after excluding the study by Camerota et al.


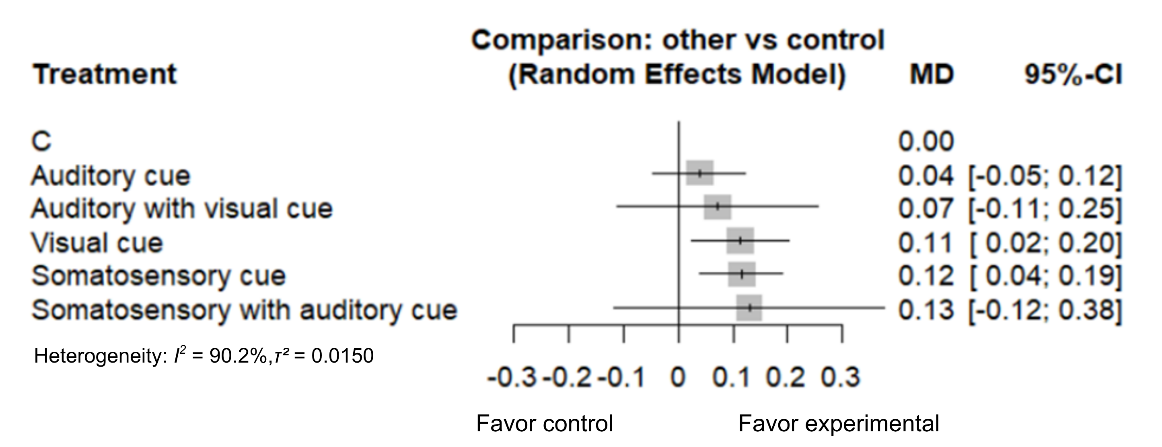


**Supplement 4:** Sensitivity analyses of cueing modalities for stride length

Figure S4. Sensitivity analysis forest plots for stride length. These plots evaluate the robustness of the network meta-analysis results by examining the impact of specific modalities and the impact of data transformation (median to mean) on overall network heterogeneity and consistency. The figures display the comparative efficacy of interventions versus control after: (a) excluding auditory cues (AC) from the network, (b) excluding visual cues (VC); (c) excluding visual cues (VC) and study by Camerota et al. from the network.

Figure S4a.


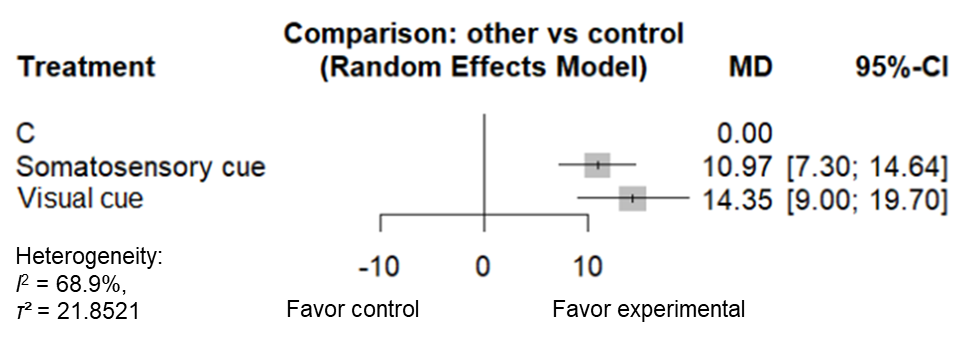


Figure S4b.


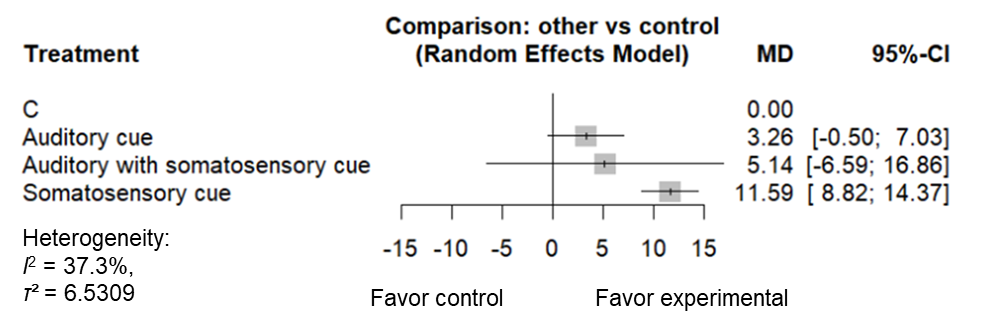


Figure S4c.


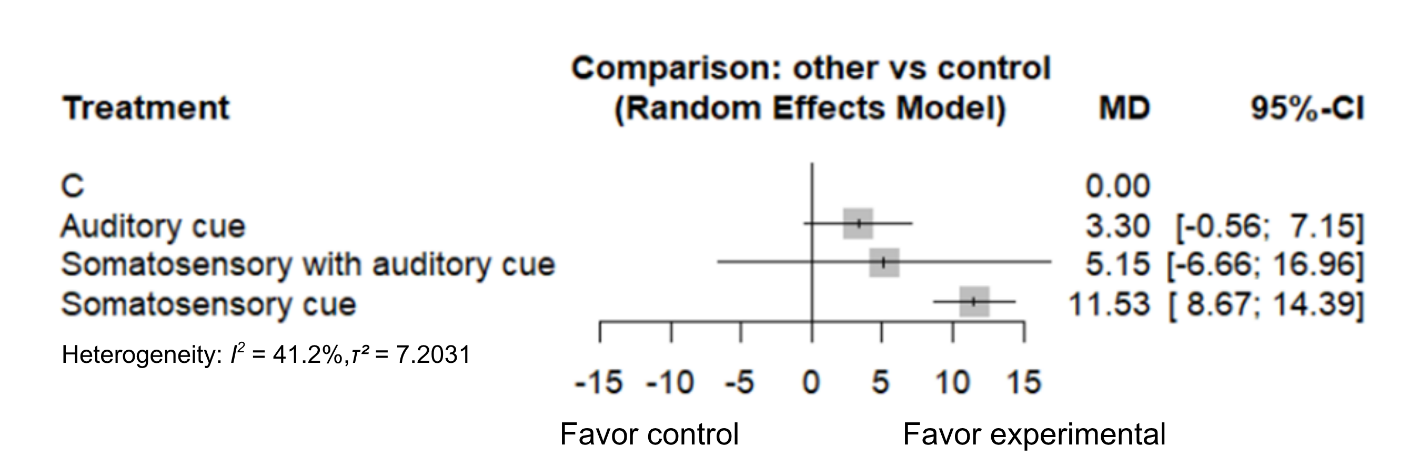

Supplement: Supplementary file 2 — Supplementary file2 (DOCX 4729 KB) [file 415_2026_13857_MOESM2_ESM.docx]
